# Preventive Medicine
†The Progress of Preventive Medicine and Sanitary Measures; being the Thurston Speech on the Mendy Commemoration at Caius College. By A. W. Barclay, M.D. Cambridge: Deighton, Bell, and Co. London: Bell and Daldy. 1856.


**Published:** 1856-10

**Authors:** 


### PREVENTIVE MEDICINE.†

The Thurston Speech of Dr. Barclay, on "The Progress of Preventive Medicine," is written in a good spirit; and if it puts forward nothing new, it contains many facts and arguments which cannot be too often repeated and impressed on the minds of earnest men.

One passage will illustrate the tone of this address:

"All advances in the practice of medicine have proceeded on a further knowledge of the causes of disease, and the exact influence of external agents upon morbid and healthy structure. That practice is utterly worthless, which in the present day goes back to the darkness of the past, and deals only with symptoms. The man who attempts to aid the operations of nature without knowing what those operations are, can only owe his success to the *vis medicatrix naturæ*. . . And it is worthy of notice, that the very persons who owe most to this kindly interference, are generally the loudest in boasting of the cures they have wrought. Medical philosophers of the present day have ceased to talk of *curing* disease."

### LUNACY LAWS AND ASYLUMS.‡

Dr. Webster, in contrasting the Lunacy Laws and the Asylums of England and France, argues that the British

---

† The Progress of Preventive Medicine and Sanitary Measures; being the Thurston Speech on the Mendy Commemoration at Caius College. By A. W. Barclay, M.D. Cambridge: Deighton, Bell, and Co. London: Bell and Daldy. 1856.

